# The effect of color type on early wound healing in farmed mink (*Neovison vison*)

**DOI:** 10.1186/s12917-017-1052-1

**Published:** 2017-05-22

**Authors:** A. Jespersen, H. E. Jensen, J. F. Agger, P. M. H. Heegaard, P. Damborg, B. Aalbæk, A. S. Hammer

**Affiliations:** 1 Kopenhagen Fur, Langagervej 60, DK-2600 Glostrup, Denmark; 2 Department of Veterinary Disease Biology, Faculty of Health and Medical Sciences, University of Copenhagen, Ridebanevej 3, DK-1870 Frederiksberg C, Denmark; 3 Department of Large Animal Sciences, Faculty of Health and Medical Sciences, University of Copenhagen, Grønnegårdsvej 8, DK-1870 Frederiksberg C, Denmark; 4 National Veterinary Institute, Technical University of Denmark, Bülowsvej 27, DK-1870 Frederiksberg C, Denmark; 5 Department of Veterinary Disease Biology, Faculty of Health and Medical Sciences, University of Copenhagen, Ridebanevej 3, DK-1870 Frederiksberg C, Denmark

**Keywords:** Mink, *Neovison Vison*, Serum amyloid a, Wound healing, Wound model

## Abstract

**Background:**

Individual differences of mink, including color type, are speculated to affect the course of wound healing, thereby impacting wound assessment and management on the farms, as well as the assessment of wounds in forensic cases. In this study, we examined the effect of color type on early wound healing in farmed mink. Full thickness excisional wounds (2 × 2 cm) were made on the back in 18 mink of the color types Brown, Silverblue and Blue Iris. Gross and microscopic pathology of the wounds was evaluated 2 days post-wounding together with degree of wound size reduction, presence of bacteria and blood analyses.

**Results:**

Pathological examination on day 2 showed the greatest mean wound size reduction in Brown mink (11.0%) followed by Blue Iris (7.9%) and Silverblue (1.6%). Bacteria were cultured from all wounds, and predominantly *Staphylococcus* species were recovered in mixed or pure culture. Histopathology from day 2 wounds showed a scab overlying necrotic wound edges, which were separated from underlying vital tissue by a demarcation zone rich in polymorphonuclear leukocytes. Fibroblasts and plump endothelial cells were more numerous in the deeper tissues. Complete blood count parameters were within normal ranges in most cases, however, the mink showed mildly to markedly decreased hematocrit and six mink of the color types Silverblue and Blue Iris showed moderately elevated numbers of circulating segmented neutrophils on day 2. There was a marked increase in concentration of serum amyloid A from day 0 to day 2 in all color types.

**Conclusions:**

We have described differences in early wound healing between mink of the color types Brown, Silverblue and Blue Iris by use of an experimental wound model in farmed mink. The most pronounced difference pertained to the degree of wound size reduction which was greatest in Brown mink, followed by Blue Iris and Silverblue, respectively.

## Background

Wounds in farmed mink are important due to their influence on animal welfare [1]. It is estimated that around 10% of mortality during growth season is caused by wounds in well-managed farms [2, 3]. The appearance, size, location and causal mechanisms of wounds may vary significantly in mink according to time of year and age [4]. Furthermore, some wound types may occur more frequently in mink of certain color types [4].

Knowledge on wound healing in mink is sparse, yet important for the optimization of wound assessment and management. Only one study has previously dealt with the healing of mink wounds. In that study, wound healing on day 12 post-wounding and later was described [5]. Forensic cases present a challenge, as the age assessment of wounds is based on knowledge of healing in other species, and not on healing in the mink per se. Due to the lack of reparative tissue, the early stages of healing are specifically challenging to assess, but offer the unique and timely presence of specific inflammatory cells, which can be studied for more accurate determination of wound age [6, 7]. Wound healing has been studied extensively in a variety of species, especially in laboratory animals used for models of human disease [8–10]. Considering the aim, different wound models have been developed to mimic specific wound types and conditions [10, 11]. Wound healing in other animal species has largely been studied to build knowledge for use in veterinary medicine regarding clinical aspects like wound assessment and therapy. From the carnivore order, wound healing in the dog and cat has been most extensively studied [12–14].

Intrinsic and extrinsic factors may influence the exact pathophysiology and course of wound healing [15]. Some fur colors of different animal species are linked to specific genetic conditions [16, 17]. In mink, the Aleutian coat color allele, resulting in a bluish grey fur color, is linked to the autosomal recessive Chédiak-Higashi syndrome, also known from humans and other species [18]. Although the syndrome seems to be more subtle in mink than in other species, and does not cause any clinical abnormalities, this color type is anecdotally more susceptible to disease than the brown wild type mink. As in other species, the condition in mink is characterized by neutrophilic changes characterized by giant granules, which have been found to be large lysosomal vesicles showing impaired degranulation [18–22]. This defect is known to affect the ability to fight bacterial infections [18, 20, 21]. We speculate that the syndrome may also affect the course of wound healing, especially in the early phases of wound healing, where inflammation and the presence of neutrophils is most pronounced [23]. Hypothetically, the neutrophilic changes would lead to a defect in the innate immune system causing inferior removal of invading microorganisms by the neutrophils [18, 20–22]. This would contribute to an increased microbial load and thereby increased susceptibility to infection. The increased microbial load would likely contribute to an altered inflammatory response due to increased recruitment of inflammatory cells to the site of injury [24–26], or there may be decreased recruitment due to defective chemotaxis [27]. Furthermore, wound closure, mediated by the replacement of lost tissue as well as wound contraction, may hypothetically be halted, or at least altered, since these mechanisms are known to be affected by the bacterial concentration and the composition of the wound microbiome [24–26]. Considering this, the Chédiak-Higashi syndrome may be of importance to the fur industry, as there may be practical implications for managing wounds in this color type.

Thus, the aim of the present study was to examine possible variations in early wound healing processes, including pathology, hematology, acute phase reaction and bacteriology, in a wound model in mink of different color types, i.e. the wild type (Brown), a blue type (Silverblue) and a pure Aleutian type (Blue Iris).

## Methods

### Animals

In total, 18 healthy commercially bred American mink of the color types Brown (*n* = 6), Silverblue (*n* = 6) and Blue Iris (*n* = 6) (Table 1) were purchased from a Danish farm and transferred to the research farm at the University of Copenhagen. The mink were kept singly in standard cages according to Danish legislation and were allowed 3 days for acclimatization upon arrival to the facility. The daily care and supervision was carried out by trained animal caretakers as well as the research participants. The feed consisted of a mink diet from a commercial feed kitchen. The mink were fed ad libitum and had free access to tap water.

**Table 1:** Animal data

*Mink number*	*Color type*	*Sex*	*Age*	*Body weight pre surgery*	*Body weight 48 h post surgery*
1	Brown	♂	4 months	1861 g	1740 g
2	Brown	♂	4 months	2806 g	2700 g
3	Brown	♂	4 months	2032 g	2000 g
4	Brown	♂	4 months	2403 g	2380 g
5	Brown	♂	4 months	2656 g	2580 g
6	Brown	♂	4 months	2483 g	2480 g
7	Silverblue	♂	4 months	2761 g	2660 g
8	Silverblue	♂	4 months	1752 g	1640 g
9	Silverblue	♂	4 months	2531 g	2440 g
10	Silverblue	♂	4 months	2636 g	2500 g
11	Silverblue	♂	4 months	1748 g	1640 g
12	Silverblue	♂	4 months	2679 g	2600 g
13	Blue Iris	♂	4 months	2490 g	2400 g
14	Blue Iris	♂	4 months	2529 g	2540 g
15	Blue Iris	♂	4 months	2285 g	2220 g
16	Blue Iris	♂	4 months	2394 g	2140 g
17	Blue Iris	♂	4 months	2284 g	2200 g
18	Blue Iris	♂	4 months	2204 g	2100 g

### Experimental procedure

The mink were weighed (Table 1) and anaesthetized with a combination of ketamine (5 mg/kg) and medetomidine (0.08 mg/kg) intramuscularly after premedication with an intramuscular injection of buprenorphine (0.02 mg/kg). Body temperature was monitored during anesthesia. The mink were surgically prepared by removing the fur at the surgical site with electrical clippers as well as by washing with chlorhexidine-cetrimide followed by 70% ethanol. A skin area of 2 × 2 cm in the dorsal midline of the thorax, at the caudal margin of the shoulder blades, was outlined with a marker pen using a plastic template which was disinfected with 70% ethanol. A new template was used for each mink. One square, full thickness excisional wound (including the panniculus carnosus) was then generated in each mink by use of scalpel and scissors. The excised skin was harvested for histological analysis. Photos of the surgical areas were taken before and after surgery, and the length and width of the wounds was measured with a caliper post-surgery. Anesthesia was reversed by intramuscular injection of atipamezole (0.4 mg/kg) and the mink were returned to their cages, receiving buprenorphine (0.02 mg/kg) intramuscularly every 6–8 h throughout the first 24 h after surgery for pain relief. The wounds were left open for 48 h with regular supervision of the mink and their feed intake.

Blood for analysis of the acute phase protein serum amyloid A (SAA) and hematology was sampled prior to surgery and at euthanasia 48 h post-surgery. The blood was sampled from the cephalic vein after immobilization of the mink in a trap. Before euthanasia, blood was sampled directly from the heart. Serum was retrieved after coagulation for 30 min at room temperature and spinning of the tubes. The samples were stored at −80 °C for up to 6 weeks until analyzed for SAA. A commercially available multispecies sandwich ELISA was applied for determination of mink SAA. This assay is based on anti-human monoclonal antibodies in a sandwich set-up [28]. Samples were analyzed according to the manufacturer’s instructions for canine SAA with the lowest sample dilution factor being 500. The detection limit of the assay was 5 mg/L (canine SAA equivalents). EDTA tubes were kept at 5 °C for a maximum of 4 h until processing for complete blood counts. Values were compared to reference ranges in mink and ferrets [29, 30].

All mink were euthanized 48 h after surgery by intracardiac injection of pentobarbital with lidocaine following anaesthetization as described above. After euthanasia, the mink were weighed (Table 1), and wounds were photographed, measured and assessed visually for appearance of scabs, undermining of skin, granulation tissue, epithelialization, edema and exudate. Swabs for bacteriological examination were taken from all wounds. The swabs were cultured on 5% calf blood agar, and incubated aerobically at 37 °C for 2 days. Subcultures of each morphologically distinct colony type were examined by matrix-assisted laser desorption/ionization time of flight (MALDI-TOF) mass spectrometry using *Escherichia coli* ATCC 8739 as reference strain and Saramis™ 3.5 for spectra interpretation. The wounds were then removed in toto and the mink necropsied [31]. Tissue samples for histopathological analysis consisted of samples from the spleen, right liver lobe, regional lymph nodes (mandibular, superficial cervical and subscapular/axillary), and adrenal glands. Control tissue consisted of skin samples from the left thigh as well as the mesenteric lymph node. All tissue samples were processed routinely after preservation in 10% neutral buffered formalin for 2 days, and stained with hematoxylin and eosin (HE). Masson trichrome stain was applied to wound tissue for visualization of collagen [32]. Histological examination with semi-quantitative assessment of epithelialization, polymorphonuclear leukocytes (PMNL), monocytes/tissue macrophages (MM), fibroblasts and endothelial cell activity was performed on all wound specimens using the scoring system given in Table 2. The area of the wounds was calculated from the measurements on day 0 and 2, respectively. The percentile reduction of the wound size from day 0 to day 2 was calculated as a measure of wound contraction:

$$ \mathrm{W}\mathrm{o}\mathrm{u}\mathrm{n}\mathrm{d}\kern0.5em \mathrm{s}\mathrm{i}\mathrm{z}\mathrm{e}\kern0.5em \mathrm{r}\mathrm{e}\mathrm{d}\mathrm{u}\mathrm{c}\mathrm{t}\mathrm{i}\mathrm{o}\mathrm{n}= 100-\left(\frac{\mathrm{wound}\kern0.5em \mathrm{a}\mathrm{r}\mathrm{e}\mathrm{a}\kern0.5em \mathrm{d}\mathrm{a}\mathrm{y}\kern0.5em  2}{\mathrm{wound}\kern0.5em \mathrm{a}\mathrm{r}\mathrm{e}\mathrm{a}\kern0.5em  d a y\kern0.5em  0}\times 100\right). $$

**Table 2:** Histological parameters and semi-quantitative scoring system for day 2 wounds

*Scale*	*Epithelialization*	*PMNL* ^*a*^	*MM* ^*b*^	*Fibroblasts*	*Endothelial cell activity*
0	No proliferation at wound edge	No or very few cells present	No or very few cells present	No or very few plump fibroblasts present	No vessels with plump endothelial cells
1	Hyperplasia/hypertrophy at wound edge	Few cells or focal pattern, ≤40% of total cellularity	≤20% of total cellularity	≥50% spindle cells, ≤50% plump fibroblasts	≤50% vessels with plump endothelial cells
2	Migration of cells over wound bed	Numerous cells present in all layers, ≥40% of total cellularity	≥20% of total cellularity	≥50% plump fibroblasts, ≤50% spindle cells	≥50% vessels with plump endothelial cells

^*a*^polymorphonuclear leukocytes; ^*b*^monocytes/tissue macrophages

For SAA, paired-samples t-test and ANOVA (test variable: pre and post-wounding concentration difference) was used to test for pre and post-wounding differences as well as differences between color types, respectively. ANOVA was used to test for differences between groups with respect to wound contraction. *P* < 0.05 was considered statistically significant.

## Results

### Clinical findings and gross pathology

**Fig. 1:**
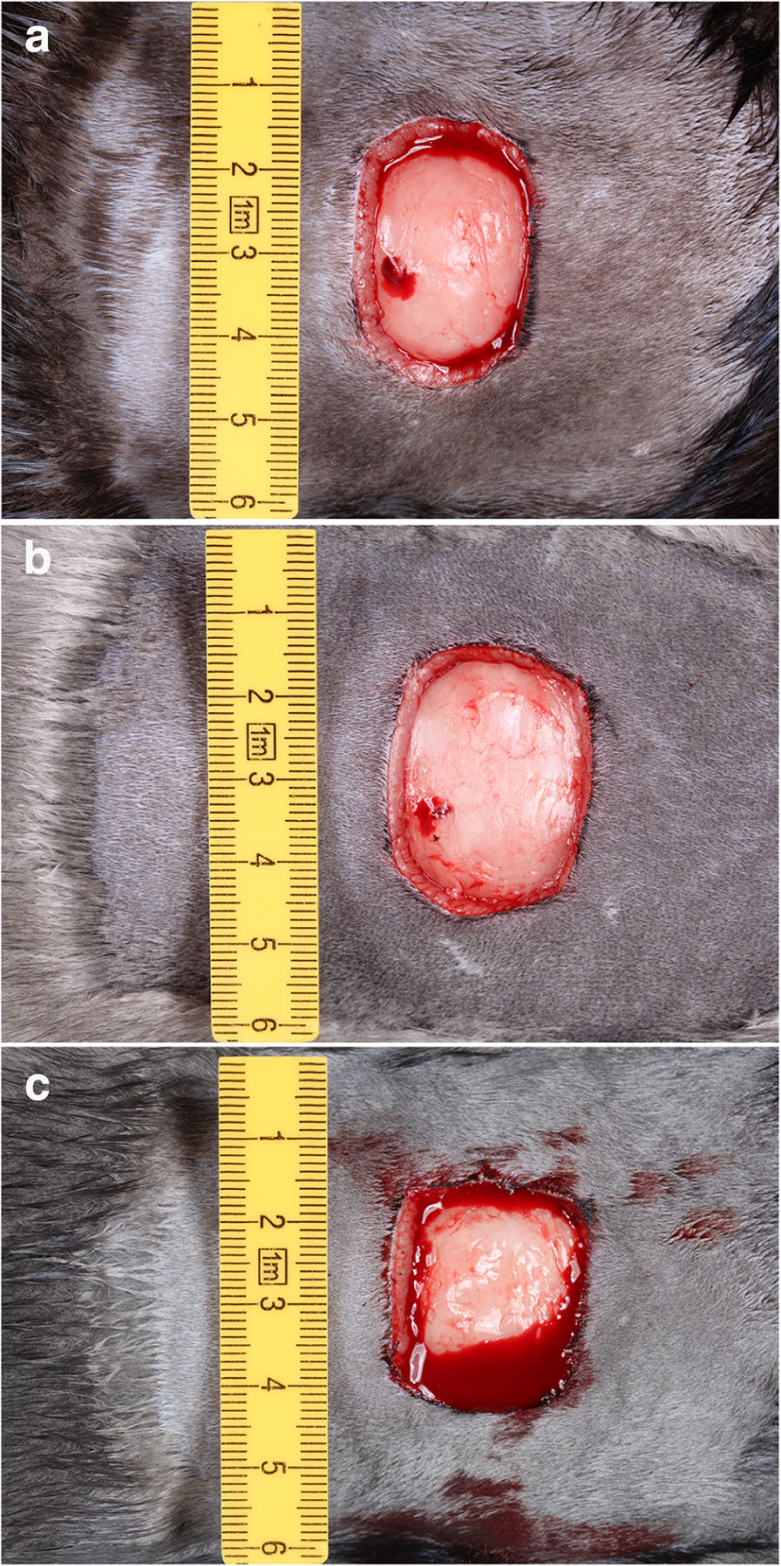
Wounds day 0, immediately post-surgery. The wounds have expanded, primarily in a latero-lateral direction, from the 4 cm^2^ template. **a**: *Brown*, **b**: *Silverblue*, **c**: *Blue Iris*. Note the more profound bleeding in the *Blue Iris* color type

Hemostasis occurred a few minutes after wound infliction in all mink except those of the color type Blue Iris, which displayed prolonged bleeding for up to 1 hour. While cutting the skin, the wound size expanded from the original 4 cm^2^ template in the latero-lateral direction (Fig. 1a-c).

**Fig. 2:**
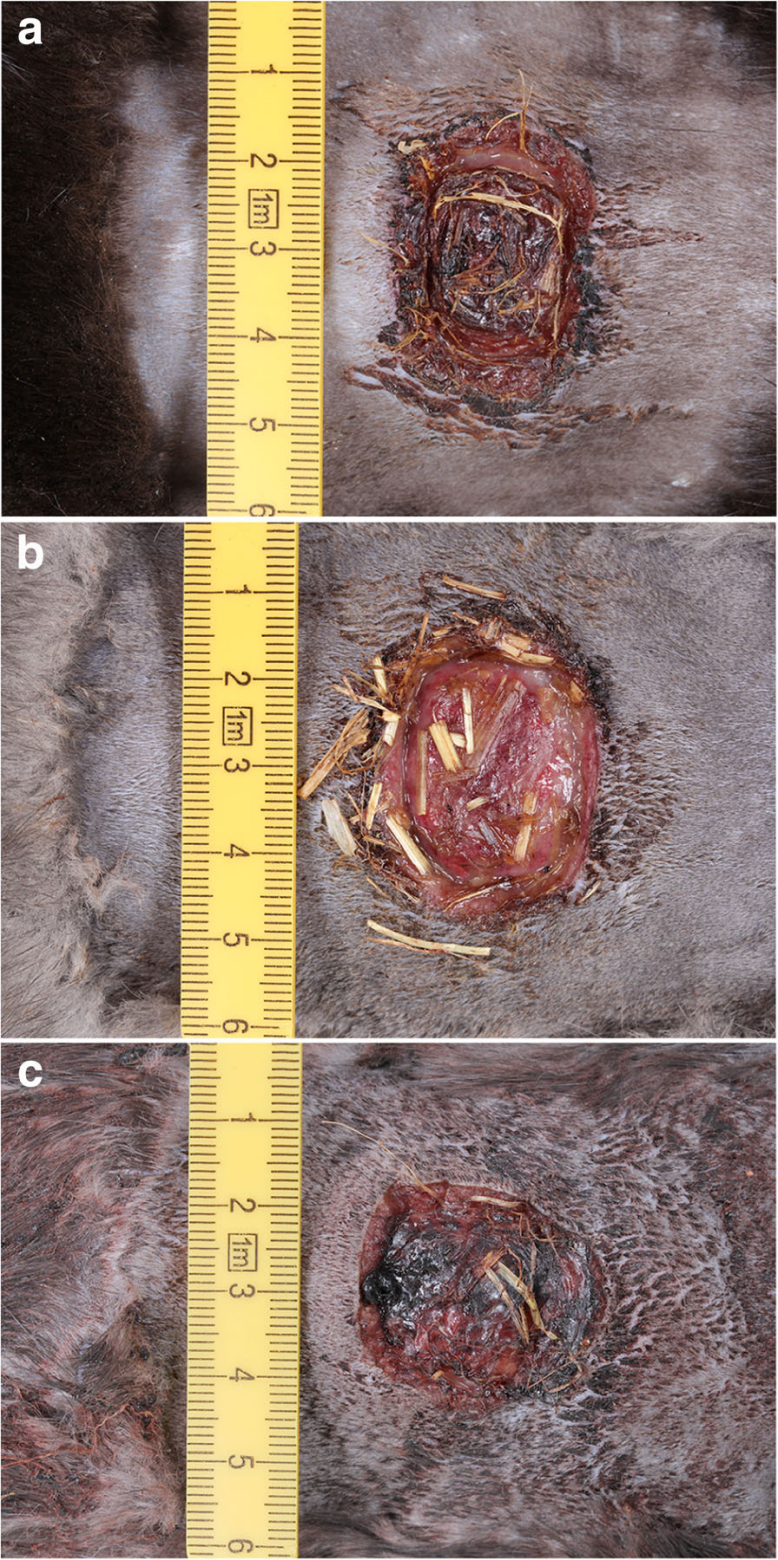
Wounds day 2, at necropsy. The wounds show varying degree of scab formation, contamination with foreign (bedding) material as well as exudation. **a**: *Brown*, **b**: *Silverblue*, **c**: *Blue Iris*. Exudate was primarily seen along the wound margins, if present (Fig. 2a)

At necropsy on day 2 post-surgery, a scab of varying thickness had formed in all cases, which partly or fully covered the wounds (Fig. 2a-c). The scab was most pronounced in mink of the color type Blue iris and Brown (Fig. 2). The cut edges were slightly thickened due to edema, and a cloudy to hemorrhagic serous exudate was seen along the wound margins in all but one mink of the Brown color type. Three mink of the color type Blue Iris showed a more purulent type of exudate, whereas the same was true in two Brown and two Silverblue mink. Furthermore, all mink showed mild to moderate splenomegaly and lymphadenopathy of regional lymph nodes.

The mink on average lost 87.4 g of body weight (average 3.8%) from day 0 to day 2. For the color types Brown, Silverblue and Blue Iris the weight loss averaged 2.7% (range −121 to −3 g), 4.7% (range −136 to −79 g) and 4.2% (range −254 to +11 g), respectively.

### Wound size

**Fig. 3:**
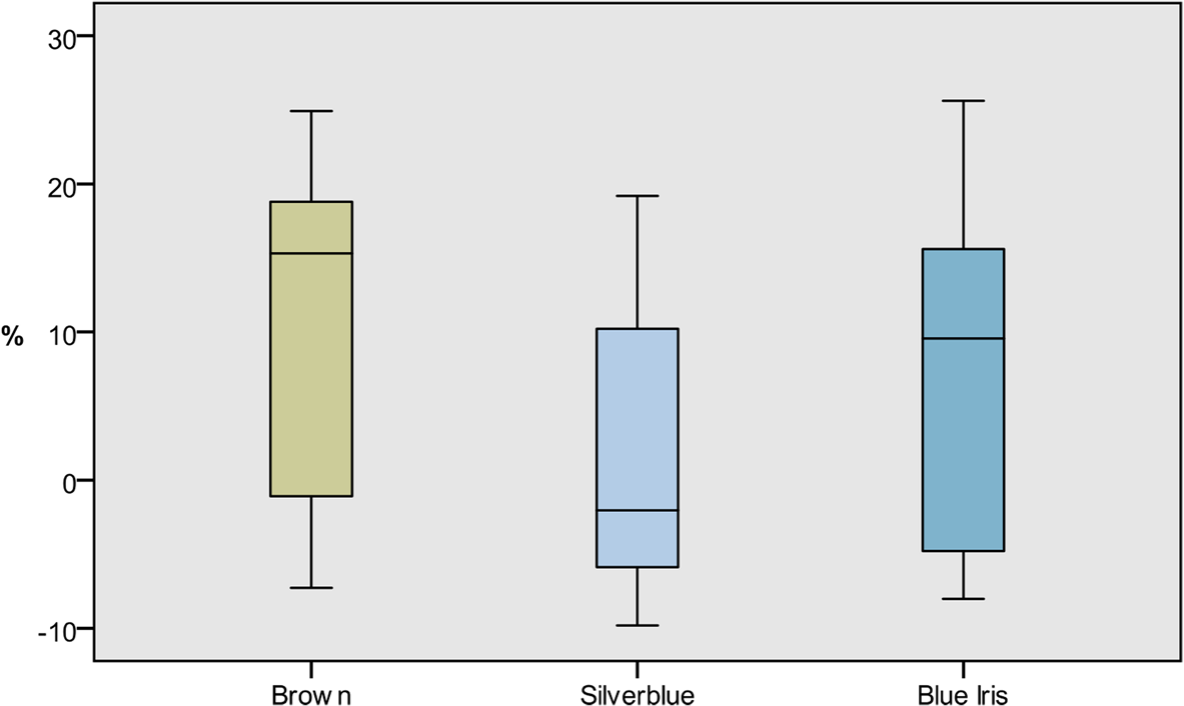
Percentage wound size reduction from day 0 to day 2 for the color types *Brown*, *Silverblue* and *Blue Iris*

The differences in wound size reduction for the three color types are shown in Fig. 3. Brown mink had the greatest mean reduction (11.0%) followed by Blue Iris (7.9%) and Silverblue (1.6%). Four mink of the Silverblue color type showed a negative reduction, i.e. an increase in wound size from day 0 to day 2, ranging from −1.1 to −9.8. For Brown and Blue Iris mink, two of each color type showed a negative reduction of −1.1 and −7.3 (Brown) and −4.8 and −8.0 (Blue Iris), respectively. The difference in wound size reduction was found not significant with ANOVA (F = 0.926; df = 2; *P* = 0.418).

### Histopathology

Day 0 skin samples showed no differences between color types and were characterized by intact skin with composite hair follicles and sebaceous glands overlying the panniculus carnosus muscle and a fatty subcutis.

**Fig. 4:**
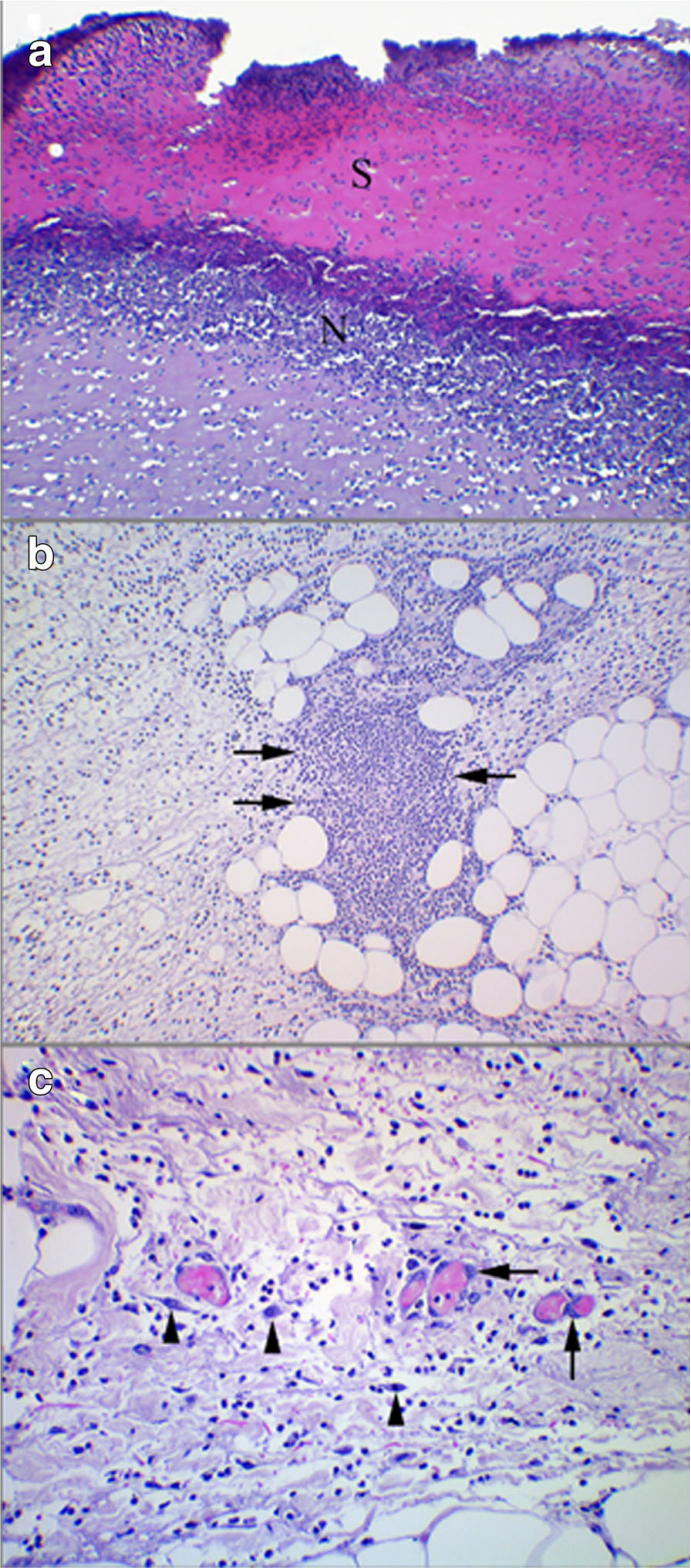
Wounds day 2. **a**: *Blue Iris* mink. A scab of non-vital tissue (S) demarcated by a zone of neutrophils (N). HE. ×100. **b**: *Silverblue* mink. Micro-abscess (*arrows*) located in edematous subcutis beneath the wound surface. HE. ×100. **c**: *Silverblue* mink. Blood filled vessels showing plumping of endothelial cells (*arrows*). Fibroblasts (*arrow heads*) in various stages of activation can be seen. HE. ×200

On day 2, the wounds were partially or completely covered by scab material consisting of intermixed necrotic tissue, debris, fibrin and erythrocytes (Fig. 4a). Bacterial colonies of cocci were found in all wound specimens as small clusters in the scab or more widespread on the wound surface. In all wounds there were also mild to moderate degrees of bleeding and deposits of fibrin as well as edema and distended lymph vessels. A demarcation zone of PMNL was separating the scab and necrotic tissue at the cut edges and in the wound bed from vital tissue (Fig. 4a). MM were present in smaller numbers. The ratio of PMNL to MM shifted towards the deeper tissues, with increasing numbers of MM relative to PMNL. Microabscesses or cellulitis were seen in the connective tissue beneath the demarcation line in 4 specimens (Fig. 4b). Plump fibroblasts were most predominant in the deeper subcutaneous tissues, however, collagen deposition was not observed in Masson trichrome stained sections. Sprouting of blood vessels was not evident, but some endothelial cells of existing blood vessels showed plumping with gap formation between adjacent cells (score 1–2, Table 2) as a sign of activity (Fig. 4c).

**Fig. 5:**
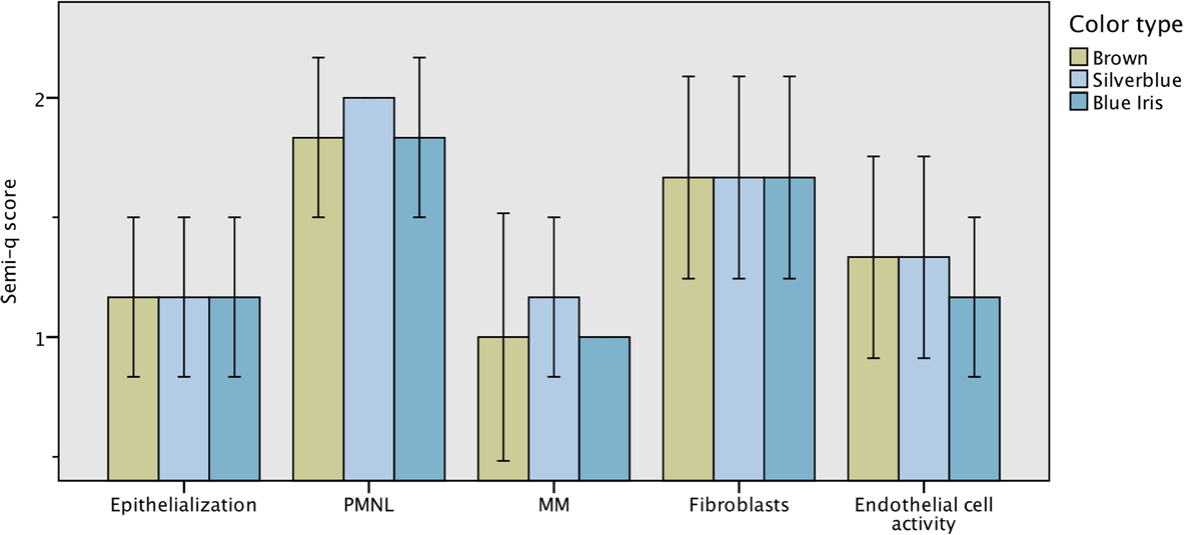
Histopathological wound examination. Semi-quantitative analysis (y-axis indicates score, see Table 2) of histological changes related to colour type. PMNL = polymorphonuclear leukocytes; MM = monocytes/tissue macrophages. +/− 2 SE

The semi-quantitative wound assessment showed minor differences between the three color types (Fig. 5). PMNL and MM scored marginally higher in the Silverblue mink than in Brown and Blue Iris, and endothelial cell activity had a slightly lower score in Blue Iris than the other color types.

Livers showed mild generalized vacuolation of hepatocytes in all mink. Furthermore, all mink showed mild to marked splenic congestion together with proliferation of periarteriolar lymphoid sheaths/lymphatic nodules and increased numbers of megakaryocyte. Regional lymph nodes were characterized by active lymphatic nodules. Some lymph nodes, however, showed a general thinning of the lymphocyte population in all (6 lymph nodes) or certain parts (8 lymph nodes) of the cut surface. Varying degrees of erythrophagocytosis was seen in 15 regional lymph nodes. In all animals, adrenal glands were approximately 50–100% larger than adrenal glands from healthy adult mink without wounds. Control samples of skin and mesenterial lymph nodes showed the normal architecture of each tissue type and were without pathological changes.

### Blood parameters

The concentration of SAA increased significantly post-wounding (paired-samples t-test; *t* = −4.748; df = 17; *P* < 0.0001). The mean basal level was 6.1 μg/ml (range 5.0–12.7 μg/ml) and increased to 1357.1 μg/ml (222 fold increase) on day 2. There was no significant differences between the three color types when comparing pre and post-wounding concentration difference in ANOVA (F = 0.739; df = 2; *P* = 0.494).

Total leucocyte, band neutrophil, lymphocyte and thrombocyte numbers were within normal ranges in all cases. The number of circulating segmented neutrophils increased from day 0 to day 2, but remained within the reference interval in all but six individuals, whose levels rose above the reference interval to between 10.1 to 13.0 × 10^9^/l. There was a drop in circulating lymphocyte numbers from day 0 to day 2, but all values remained within the reference interval. Erythrocyte numbers decreased from day 0 to day 2. Five mink, all of the color type Blue Iris, had moderate to severe regenerative to non-regenerative anemia on day 2 characterized by a decrease in total erythrocyte numbers, hematocrit and hemoglobin concentration. Moreover, all Blue Iris mink showed vesicle-like inclusion bodies of varying size in a proportion of the circulating neutrophils.

### Bacteriology

Bacteria were cultured from all wounds on day 2. In 9 wounds, the bacterial isolates were from the *Staphylococcus intermedius* group (SIG) which was found as monoculture in 2 wounds and in mixed culture in 7 wounds. The bacterial isolates are given in Table 3. There was no relationship between color type and wound bacterial species.

**Table 3:** Bacterial isolates from all day 2 wounds

*Mink number*	*Brown*	*Silverblue*	*Blue Iris*
1	SIG monoculture	SIG + *Streptococcus canis*	*Streptococcus equi* monoculture
2	SIG monoculture	SIG + *Streptococcus dysgalactiae*	*Streptococcus canis* monoculture
3	*Staphylococcus schleiferi* monoculture	SIG + *Streptococcus* sp.	SIG + *Pasteurella canis*
4	*Streptococcus dysgalactiae* monoculture	SIG + *Corynebacterium ulcerans*	SIG + *Corynebacterium ulcerans*
5	SIG + unidentified sp.	*Bacillus cereus* + *Streptococcus dysgalactiae*	*Staphylococcus schleiferi* + *Streptococcus equi*
6	*Staphylococcus schleiferi* + *Streptococcus* sp.	*Corynebacterium ulcerans* + unidentified sp.	*Staphylococcus schleiferi* + unidentified sp.

SIG *Staphylococcus intermedius* group

## Discussion

The most pronounced effect of color type on wound healing was on the differences in wound size reduction from day 0 to day 2, which showed a greater contractile ability in Brown mink than in Blue Iris and Silverblue mink (Fig. 3). The differences were, however, found not significant and thus represent tendencies. The reason for the differences may be associated with genetic factors modulating the composition of the skin or the body’s ability to raise an immunological response and react to tissue damage [33]. Our hypothesis that Aleutian mink would show delayed or inferior wound healing compared to other color types was only partly confirmed, as the Silverblue mink in this study showed slower wound size reduction than the Aleutian Blue Iris mink. This indicates that factors associated with healing in other color types may be equally important determinants of wound healing and therefore of importance to the fur industry.

The 48 h duration of the study was chosen because of the hypothetical presence of both early and late acute stage inflammatory cells [23]. The minor differences in average scores of the semi-quantitative histological analysis indicate that color type does not influence these important histological parameters significantly. It can thus be assumed, that the histological parameters studied can be used as designators of wound age regardless of color type, at least in the early stages of healing. The histopathological appearance of the mink wounds was easily comparable to findings in other mammals, as expected. Our wound model is comparable to studies of wound healing in the dog and cat [12–14]. Mink are closer phylogenetic relatives to dogs than to cats [34], but show high resemblance to the cat karyotype [35]. Looking at early wound healing in these carnivores, there seems to be greater degree of edema formation in the dog than in the cat [14]. Early wound healing in mink seems therefore most comparable to wound healing in the cat, as we saw only slight edema formation on day 2 post-wounding in our study.

The mink showed loss of body weight from day 0 to day 2. Apart from the wounds, the mink were healthy and were in their growth period, so weight loss is considered abnormal. We speculate that the weight loss was the result of reduced feed intake post-wounding; however, it is not certain whether this was due to stress or fatigue from handling, an effect of anesthesia, or the presence of wounds. The enlargement of adrenal glands was apparently caused by hyperplasia and/or hypertrophy of the adrenal zona fasciculata and medulla. Chronic stress can lead to adrenal enlargement [36] and even acute heat stress has been shown to cause a slight increase in adrenal mass in rats [37]. The observed adrenal hyperplasia may indicate that stress was involved, but adrenal changes related to stress has apparently not been studied in the mink. Including negative controls in the form of a handled, non-wounded group as well as a sham-treated group of mink would be warranted in future studies in order to more accurately explain the cause of adrenal enlargement as well as weight loss. Apart from the effect on adrenal morphology, stress is known to influence the wound healing process by causing a delay in tissue repair [38].

Splenomegaly and lymphadenopathy of regional lymph nodes reflect the microscopic findings of splenic congestion and proliferation of white pulp and lymph node activity, which was not a surprising finding given the wounds and resulting inflammatory response. In our experience, the spleen of mink is very reactive in the case of injury; however, splenic congestion and enlargement may furthermore be the result of the barbiturate used for euthanasia of the mink [39]. Again, negative controls would have been beneficial in order to differentiate between the possible mechanisms of splenic enlargement.

An interesting finding was that all five mink exhibiting anemia on day 2 were of the color type Blue Iris. All mink showed a decrease in hematocrit on day 2 compared to day 0, which may be due to blood sampling. However, adding the blood loss from the prolonged bleeding in Blue Iris mink likely explains why anemia was only observed in this color type. The blood loss was, however, not quantified. The Chédiak-Higashi syndrome is linked to the Aleutian trait of Blue Iris mink, and is known to cause delayed hemostasis and increased tendency for wound infection due to a defective platelet aggregation function and a lower resistance to infection [18, 21, 40]. The finding of inclusion bodies in circulating neutrophils of Blue Iris mink in this study is characteristic of the Chédiak-Higashi syndrome. The inclusion bodies represent enlarged lysosomes showing impaired ability to fuse with phagosomes containing foreign particulates like invading bacteria engulfed by the cell [19–22]. Three mink of the color type Blue Iris showed purulent exudate, strongly indicating wound infection, whereas the same was true for two Brown and two Silverblue mink. However, these numbers are not enough to conclude, that mink carrying the Aleutian trait are more susceptible to wound infection.

The function and expression of SAA and its use as a sensitive marker of inflammation has been studied in many species including the mink [41–46]. In accordance with results from our previous study on early wound healing in mink, we found a significant mean increase of SAA concentration from day 0 to day 2 (222 fold). There was, however, no effect of color type on the SAA response.

The isolation of a large proportion of bacteria from the *Staphylococcus intermedius* group (SIG) from the wounds is in accordance with findings in our previous study of experimental wounds. SIG comprises *Staphylococcus intermedius, Staphylococcus pseudintermedius* and *Staphylococcus delphini* group A and B [47], which could not be distinguished by MALDI-TOF MS. However, the isolates are most likely *Staphylococcus delphini* Group A [48], which has been reported to be an opportunistic pathogen in mink [48–50]. Whether the isolated bacteria were part of an early infectious process or just wound contaminants was not evident, though the presence of microabscesses and cellulitis in some wound specimens may indicate spreading infection. Moreover, a relationship between color type and microbiological findings could not be deduced. The presence of bacteria in the wounds is, however, likely to influence the process of wound healing [24–26].

## Conclusions

To conclude, we have studied the process of early wound healing in a wound model in farmed mink of different colors. The most striking finding was the difference in contractile ability, where Brown showed the greatest wound size reduction followed by Blue Iris and Silverblue. Only minor differences in histopathological parameters were found between the color types. Bacteria, mainly SIG isolates, were cultured from all wounds; however, the significance of the isolates is unknown. Finally, we found a highly significant rise in SAA from day 0 to day 2 indicating a profound effect of wounds on the acute phase response in mink. The results indicate that color type may affect speed of wound healing, which may have implications for clinical/practical wound management. Furthermore, the marked rise in SAA post-wounding suggests that wounds have a significant effect on the mink immune system and that wound healing is a highly prioritized physiological process. Future studies of the effect of color type and other factors on wound healing including late stage healing and breaking strength of healed skin is considered relevant.

## Abbreviations

ANOVA: Analysis of variance
EDTA: Ethylene-diamine-tetra-acetic acid
ELISA: Enzyme-linked immunosorbent assay
HE: Hematoxylin and eosin
MALDI-TOF: Matrix-assisted laser desorption/ionization time of flight
MM: Monocyte/tissue macrophage
PMNL: Polymorphonuclear leukocyte
SAA: Serum amyloid A
SIG: *Staphylococcus intermedius* group
